# Evaluation of the primary stability in dental implants placed in low density bone with a new drilling technique, Osseodensification: an in vitro study

**DOI:** 10.4317/medoral.24231

**Published:** 2020-10-09

**Authors:** Javier Barberá-Millán, Carolina Larrazábal-Morón, Juan José Enciso-Ripoll, Esteban Pérez-Pevida, David Chávarri-Prado, María Dolores Gómez-Adrián

**Affiliations:** 1 Doctoral School. Catholic University of Valencia San Vicente Mártir, Valencia, Spain; 2 Department of Surgery and Oral Implantology, Faculty of Medicine and Dentistry, Catholic University of Valencia, Valencia, Spain; 3 Department of Surgery, Faculty of Medicine, University of Salamanca, Salamanca, Spain; 4 Faculty of Health Sciences, Miguel de Cervantes European University, Valladolid, Spain; 5 Department of Surgery and Medical-Surgical Specialties, University of Oviedo, Oviedo, Spain.

## Abstract

**Background:**

Primary stability is an important key determinant of implant osseointegration. We investigated approaches to improve primary implant stability using a new drilling technique termed osseodensification (OD), which was compared with the conventional under-drilling (UD) method utilized for low-density bones.

**Material and Methods:**

We placed 55 conical internal connection implants in each group, in 30 low-density sections of pig tibia. The implants were placed using twist drill bits in both groups; groups Under Drilling (UD) and Osseodensification (OD) included bone sections subjected to conventional UD and OD drilling, respectively. Before placing the implants, we randomized the bone sections that were to receive these implants to avoid sample bias. We evaluated various primary stability parameters, such as implant insertion torque and resonance frequency analysis (RFA) measurements.

**Results:**

The results showed that compared with implants placed using the UD technique, those placed using the OD technique were associated with significantly higher primary stability. The mean insertion torque of the implants was 8.87±6.17 Ncm in group 1 (UD) and 21.72±17.14 Ncm in group 2 (OD). The mean RFA was 65.16±7.45 ISQ in group 1 (UD) and 69.75±6.79 ISQ in group 2 (OD).

**Conclusions:**

The implant insertion torque and RFA values were significantly higher in OD group than in UD. Therefore, compared with UD, OD improves primary stability in low-density bones (based on torque and RFA measurements).

** Key words:**Osseodensification, primary stability, low density bone, RFA.

## Introduction

Currently, primary implant stability is considered a prerequisite for osseointegration. Primary stability is a static and purely mechanical parameter, which is determined at the time of implant placement and is associated with resistance or friction between the bone and the implant upon insertion (1-4). Primary stability can be affected by multiple factors, including recipient bone density, implant design, surgical technique, or operator experience (5-8).

Numerous techniques have been proposed over the years to measure primary stability; currently, implant insertion torque and resonance frequency analysis (RFA) measurements are the most commonly accepted biomechanical parameters used for this purpose (1,2,7-9). Both parameters predict primary implant stability; however, they differ in their approach. Implant insertion torque measures the resistance encountered during implant advancement in the apical direction. RFA measurement is based on detection of the natural frequency of vibration of the implant within the bone, which depends on the rigidity of its connection with the bone and determines its degree of micromovement (1,10). Studies have reported that when the implant micromovement exceeds a specific threshold (50–150 µm), fibrous encapsulation prevails over osseointegration (10). Notably, the main difference between the aforementioned parameters is that implant insertion torque can only be recorded at the time of implant placement; therefore, stability monitoring or tracking over time is not possible. In contrast, RFA enables long-term monitoring of stability parameters (2,9-12).

According to the Lekholm & Zarb classification proposed in 1985 (13), type IV low-density bone, characterized by a fine layer of cortical bone (occasionally absent) surrounding a low-density trabecular bone core, is usually observed in the posterior maxilla. It is difficult to achieve adequate primary stability for osseointegration with implants placed into this type of bone; therefore, it is important to consider modifications to the drilling technique, operator experience, and implant macrodesign in this clinical setting (1,4,6-8).

Hole drilling (HD) is the main surgical technique used to perform ostectomy and to create the implant bed. However, HD in low-density (type IV) bones is associated with low primary stability for dental implant osseointegration (7,14-16). Therefore, several techniques have been described to improve osseointegration. Under-drilling (UD) refers to the process of preparing an implant bed with a diameter that is considerably smaller than the implant diameter, which thereby improves primary stability (4,7,17), although such stability is often insufficient. The bone expansion technique using expansion osteotomes to create the bone bed was an alternative attempted to improve primary implant stability (4,6,14-16). Osteotomes enable condensing of bone trabeculae, which improves peri-implant bone density rather than removing bone by drilling, with consequently improved primary stability (18).

However, osteotomes used for osseocondensation usually cause greater surgical trauma secondary to the impact delivered by the hammer. OD is a novel implant preparation technique that improves the primary stability of implants placed in low-density bones by overcoming the drawbacks of the aforementioned techniques (7,19-21). This approach combines the two previously described techniques, using OD drill bits, which are rotated in a counterclockwise direction at a speed of 1200 revolutions per minute (rpm), with abundant irrigation to cause bone compaction both apically and laterally against the walls of the implant bed to improve primary stability by increasing the percentage of bone-implant contact (20,21).

In this *in vitro* experimental study, we compared the primary stability of implants placed using the OD vs. UD technique based on implant insertion torque and RFA measurements. Additionally, we investigated the association between these parameters.

## Material and Methods

- Sample selection

**Figure 1:**
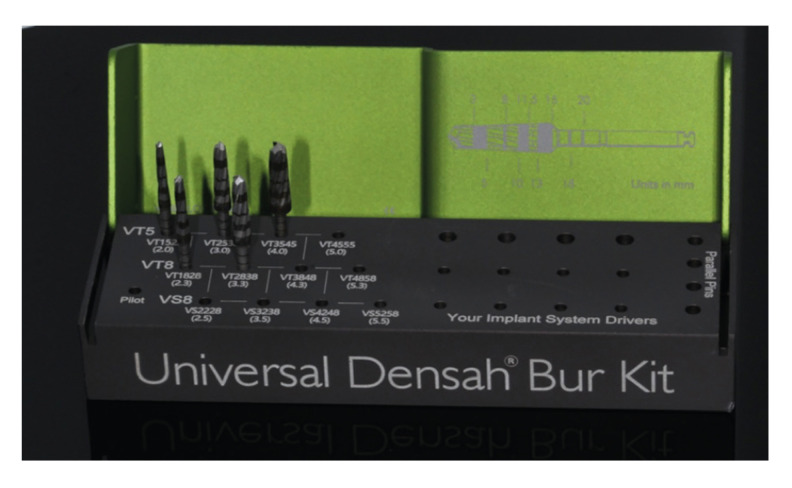
Densah® burs, Versah, LLC, Jackson MI, USA.

In this study, we used 110 Klockner Vega internal connection bone-level implants (Soadco, Escaldes-Engordany, Andorra) measuring 4 mm in diameter and 10 mm in length. The implants were categorized into a control group (group 1, 55 implants), which were placed using the UD technique and a test group (group 2, 55 implants), which were placed using the OD technique for which we used drill bits (Densah® burs, Versah, LLC, Jackson MI, USA) (Fig. 1).

- Sample preparation

Osteotomies were performed in 30 coronal sections of frozen fresh pig tibias (Maxylar®, Girona, Spain) with mechanical properties resembling those of low-density human maxillary bone (type D4, based on the Lekholm & Zarb classification) (13). The samples were preserved under vacuum in a thermal insulation container with dry ice with the temperature maintained at -78°C to preserve tissue integrity and characteristics. Subsequently, the samples were thawed for 5 hours at room temperature before study commencement. The groups were randomized using envelopes and 55 implants were assigned to each group.

**Figure 2:**
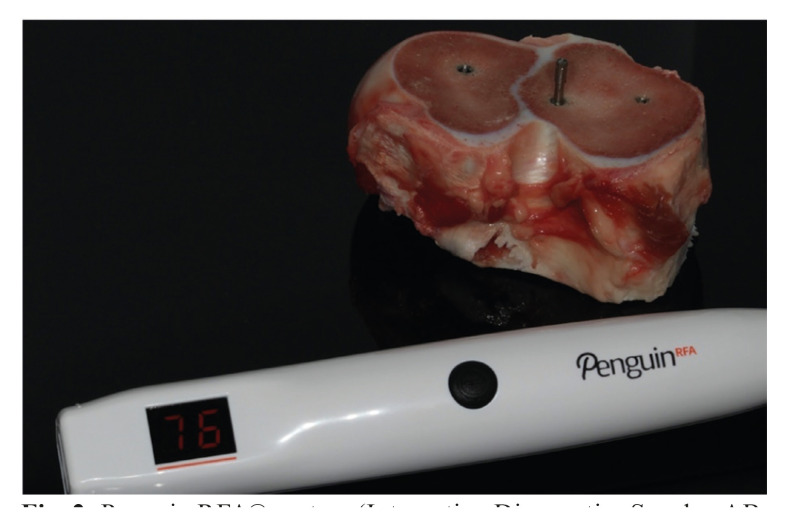
Penguin RFA® system (Integration Diagnostics Sweden AB, Göteborg, Sweden).

We placed 3 implants in each section. Among the beds of these implants, two were created using OD or UD to ensure that they were always in the same location in the coronal section. No bone section underwent three osteotomies from the same study group (Fig. 2).

The preparation of the implant beds and implant insertion were performed by the same operator, who also recorded the torque and RFA measurements.

- Drilling systems

In group 1, we used the specific drilling sequence recommended by the manufacturer (Soadco, Escaldes-Engordany, Andorra) for an implant measuring 4 mm in diameter. We did not use the last drill bit and performed UD of the bed. Therefore, the following drilling sequence was used: drilling to a depth of 10 mm using the initial drill bit (0–2.35 mm) at 1200 rpm, a second pilot drill bit (2.35 mm) at 600 rpm, a third drill bit (2.8 mm) at 600 rpm, and a final drill bit (3.3 mm) at 600 rpm.

In group 2, we used the specific drilling sequence recommended by the manufacturer (Versah, Jackson, MI, USA) for soft bone. Drilling was performed using the OD protocol recommended for implants measuring 3.5 mm. Counterclockwise drilling was performed at 1200 rpm with all drill bits, along the length of the implant with abundant saline irrigation. The following protocol was followed: The initial drill bit was first used (0–2.35 mm), followed by the first OD drill bit (2.0 mm), second (2.3 mm), third (3.0 mm), and final (3.3 mm) drill bits.

- Implant insertion and stability measurements

Primary stability was measured using the following parameters: the implant insertion torque of each implant was recorded using a calibrated Implantmed dental implant motor (W&H®, Bürmoos, Austria). Implant insertion commenced at 5 Ncm of torque, gradually increasing this value in 5 Ncm increments until complete implant insertion was achieved with the device placed at the epicrestal level (22). When the implant insertion torque was >50 Ncm, its insertion was completed using a ratchet wrench, recording the value of the result in these cases as “>50 Ncm”.

After implant placement, we recorded the implant stability quotient (ISQ) values using the Penguin RFA® system (Integration Diagnostics Sweden AB, Göteborg, Sweden) (Fig. 2). The ISQ was measured at 4 sites to simulate the mesial, distal, vestibular/buccal and palatal/lingual positions. A MultiPegTM (Integration Diagnostics Sweden AB, Göteborg, Sweden) was mounted onto the implant using its driver and screwed into place with a torque wrench and a screwdriver using 68 Ncm of force, as recommended by the manufacturer.

- Statistical analysis

The sample was analyzed using the “R” package (programming language and free software environment for statistical computing and graphics), indicated for data analysis in the field of the health sciences, using the mean measurements recorded in each case as ISQ value of the implant. Therefore, we obtained a total sample of 110 ISQ and 110 torque values.

Using these data, we used the Student's t test to compare the ISQ and torque values obtained with each technique independently. Subsequently, we performed sigmoid regression to represent the association between the torque and ISQ values of each technique using a curve (torque values) and points (ISQ values).

A *p* value <0.05 was considered statistically significant.

## Results

The results showed that compared with implants placed using the UD technique, those placed using the OD technique were associated with significantly higher primary stability. The mean insertion torque of the implants was 8.87±6.17 Ncm in group 1 (UD) and 21.72±17.14 Ncm in group 2 (OD). The mean RFA was 65.16±7.45 ISQ in group 1 (UD) and 69.75±6.79 ISQ in group 2 (OD) (Table 1). Table 2 shows significant intergroup differences in the ISQ (*p*=0.001) and implant insertion torque (*p*=0.000) values (Table 2).

**Figure 3:**
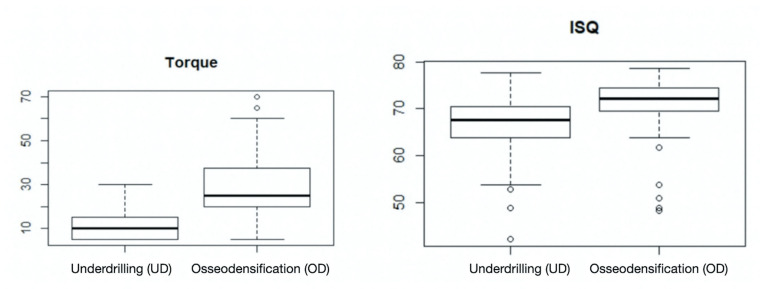
Sigmoid regression analysis between ISQ and torque.

Sigmoid regression analysis showed that ISQ values gradually increased to 75.6 Ncm with increasing torque (Fig. 3). This value was the limit beyond which any increase in torque was not associated with a corresponding increase in ISQ values.

**Figure 4:**
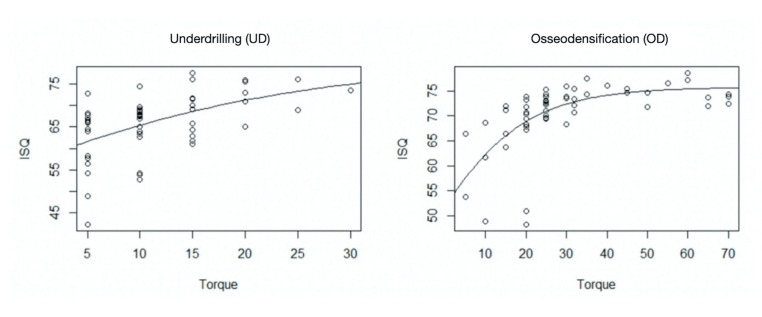
Association between torque and ISQ values.

We investigated the association between torque and ISQ values in implants placed using the UD technique and observed that the difference between torque and ISQ values decreased with increasing torque (Fig. 4).

**Table 1:**
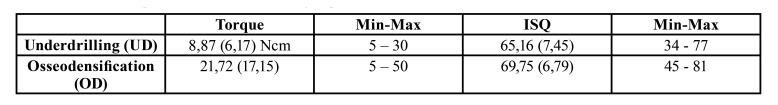
Insertion torque and ISQ values for both groups.

**Table 2:**
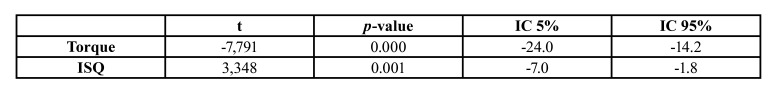
Signification of diferences of torque and ISQ values.

Notably, this difference decreased further in implants placed using the OD technique. The association between torque and ISQ values was represented using the pseudo r2 value, which was higher in the OD (pseudo r2=0.420) than in the UD group (pseudo r2=0.272), indicating a better fit, in addition to a lower standard error in the OD (standard error=6.10) than in the UD group (standard error=4.99).

## Discussion

In this study, we used sections of pig tibia to simulate the mechanical characteristics of low-density human maxillary bone. Previous *in vitro* studies have used bones with similar characteristics, such as corticotomized porcine (14,17) and bovine (9,23,24) ribs, as well as femoral heads from human cadavers (6). This study model was used instead of the rib model to avoid the process of removing the cortical bone that surrounds trabecular bone. It is important to remove the cortical bone because presence of cortical bone in the coronal zone interferes with accurate evaluation of primary stability, as reported by previous studies (9).

Despite the lack of homogeneity in all bone sections investigated (reported by studies performed using polyurethane blocks (1,4,25-28)), it is reasonable to conclude that the sample size of 55 implants per group and their randomization reduced the likelihood of bias in this study. Moreover, by ensuring preservation of tissue integrity and tissue properties, this model successfully simulated a real-world clinical situation. Our results highlight the effect of the drilling technique used to perform the osteotomy on the primary stability of dental implants, measured in terms of the insertion torque and ISQ values of the implant. Based on the results of primary stability, all implants (inserted using the UD or OD technique) can be subjected to immediate loading, according to the literature (5,29).

These results corroborate the findings of several previous studies. Similar to our study, Huwais *et al*. (7) placed 72 implants in 12 porcine tibias and compared the implant insertion torque of implants placed using HD and OD (36 implants in each group). Their results showed significantly higher implant insertion torque values in the OD group than in the HD group, indicating that the OD drilling protocol significantly improves primary stability.

Our results also concur with those reported by Santamaria-Arrieta *et al*. (9) [2016]. These authors placed 32 implants in 8 corticotomized veal rib blocks to simulate implant insertion in purely trabecular bone and reported results similar to those observed in our study (with regard to both torque and ISQ values). In vitro studies were performed in porcine ribs without cortical bone by Moon *et al*. (17) and Rastelli *et al*. (15) in 2010 and 2014, respectively. In the former study, primary stability was investigated based on RFA measurements of 120 implants placed using three different drilling techniques, such as HD, UD, and over-drilling in ribs with (n=60) and without (n=60) cortical bone. The ISQ values of the group without cortical bone subjected to the UD technique match the values obtained in our study. In the latter study, primary stability was investigated based on RFA measurements of implants placed using piezo-surgery, HD, UD, use of bone expanders, and osteodistraction; the results of this study were similar to those obtained in the UD group in our study.

A study performed by Chávarri-Prado *et al*. (1) in 2020 reported the placement of 40 implants identical to those used in our study. These implants were placed in polyurethane blocks with osteotomy using HD. RFA measurements were obtained using the Penguin RFA® system (Integration Diagnostics Sweden AB, Göteborg, Sweden) similar to the method used in our study; however, torque was recorded using a calibrated torque wrench (as opposed to measurement of torque using a surgical motor in our study). Interestingly, the torque and ISQ values reported by these authors are similar to those observed in our study.

Karl *et al*. (4) [2018] used polyurethane blocks and compared three different techniques to prepare the bone bed (this study did not use OD). The authors investigated the role of HD, UD, and bone expansion with osteotomes. Despite the use of different materials, insertion torque values for implants placed using HD were similar to those observed in our study.

Numerous *in vivo* human studies have investigated the association between drilling techniques and primary stability in low-density bones (2,3,16,22,30,31). Studies reported by Lee *et al*. (31) [2010] and Sadeghi *et al*. (16) [2008] are the most representative and comparable to our study with regard to the method used. The authors compared HD with bone expansion using osteotomes and observed higher ISQ values than those observed in our study, which is attribuTable to the fact that their study sample included implants placed in bone sections with cortical bone, which improves primary implant stability (1,9).

The ISQ values obtained with the use of the OD technique cannot be compared with any prior study because to date, no reports in the available literature have described this novel technique.

## Conclusions

Based on the results of this study, we conclude that compared with the conventional HD technique, the OD technique improves the primary stability of dental implants in low-density bones, based on implant insertion torque and RFA measurements. However, further clinical studies are warranted to confirm these findings and to support the use of this innovative drilling technique in low-density bones.
